# Fulvestrant 500 milligrams as endocrine therapy for endocrine sensitive advanced breast cancer patients in the real world: the Ful500 prospective observational trial

**DOI:** 10.18632/oncotarget.17262

**Published:** 2017-04-20

**Authors:** Luca Moscetti, Maria Agnese Fabbri, Clara Natoli, Patrizia Vici, Teresa Gamucci, Isabella Sperduti, Laura Iezzi, Elena Iattoni, Laura Pizzuti, Carmine Roma, Angela Vaccaro, Giuliana D’Auria, Mariella Mauri, Lucia Mentuccia, Antonino Grassadonia, Maddalena Barba, Enzo Maria Ruggeri

**Affiliations:** ^1^ Division of Medical Oncology, AUSL Viterbo, Belcolle Hospital Strada Sammartinese, 01100 Viterbo, Italy; ^2^ Department of Medical, Oral and Biotechnological Sciences and CeSI-MeT University G. D'Annunzio, Chieti-Pescara, 66100 Chieti, Italy; ^3^ Division of Medical Oncology 2, Regina Elena National Cancer Institute, 00144 Roma, Italy; ^4^ Medical Oncology Unit, ASL Frosinone, 03100 Frosinone, Italy; ^5^ Biostatistics Unit, Istituto Regina Elena, 00144 Rome, Italy; ^6^ Medical Oncology Unit, SS. Annunziata Hospital, 66100 Chieti, Italy; ^7^ Division of Oncology, Azienda Ospedaliera San Giovanni Addolorata, 00184 Rome, Italy; ^8^ Department of Oncology and Haematology, Azienda Ospedaliero Universitaria Policlinico di Modena, 41124 Modena, Italy

**Keywords:** fulvestrant, metastatic breast cancer, endocrine therapy, endocrine resistance

## Abstract

The observational prospective trial herein presented aimed at evaluating the efficacy of fulvestrant 500 mg in the treatment of endocrine sensitive advanced breast cancer patients from the real world setting. The primary end point was clinical benefit rate (CBR). Secondary end points were overall survival (OS), progression free survival (PFS) and tolerability. One hundred sixty three patients were enrolled. At a median follow up of 20 months, the 61% of patients reached CBR, whose median duration was 10.8 months. Median PFS and OS were 7 and 35 months, respectively. Endocrine sensitive patients showed better PFS and OS. No relevant toxicity appeared when analyzing safety data. In multivariate analysis, visceral involvement, endocrine sensitivity and previous endocrine therapy were prognostic factor for PFS, whereas endocrine sensitivity and metastasis at diagnosis had prognostic relevance for OS. Estrogen receptor expression >50%, single metastatic site, and no prior endocrine therapy for advanced disease were predictive of CBR. In this prospective trial, fulvestrant 500 mg appeared to be a safe and active treatment and confirmed its efficacy in the daily clinical practice. A high percent expression of estrogen receptors (above 50%) was associated with higher CBR. Treatment was very well tolerated. Endocrine sensitivity had a major impact on treatment outcome. As expected, patients who had received first-line endocrine therapy for advanced disease exhibited worse outcome and a lower CBR.

## INTRODUCTION

Breast cancer is the most common cancer in women and its incidence rises in postmenopause [1]. In postmenopausal women, hormonal-receptor positive breast cancer is the most frequent subtype. Notwithstanding the increasingly common diagnosis at an early stage and the goals achieved in the adjuvant setting, about one third of these patients is expected to relapse [2, 3]. Endocrine therapy is the mainstay of treatment in hormone receptor positive HER2 negative postmenopausal advanced-breast cancer patients. The therapeutic armamentarium has greatly enlarged and currently includes a notable number of drugs, mainly, but not exclusively, represented by the selective estrogen receptor modulators (SERM), steroidal and non-steroidal aromatase inhibitors (AIs) and selective ER down-regulators (SERD) [4]. In first line, the use of AIs was associated with higher response rates and longer time to treatment progression compared to tamoxifen [5–7]; unfortunately, most of the patients will experience disease progression while on or after AIs, thus requiring further therapeutic options. Fulvestrant is a 7α-alkylsulfinyl analog of 17β-estradiol that works as a competitive estrogen receptor (ER) antagonist but, differently from tamoxifen, it shows no agonist activity [8]. Throughout binding to its ligand, fulvestrant induces the rapid degradation of the ER and leads to the reduction of its intracellular levels; moreover, ER not only lacks of intrinsic estrogen-agonist effects, but also induces a down-regulation of the progesterone receptor (PgR), thus blocking the proliferative signaling from hormonal-activated pathways [9]. The clinical effectiveness of fulvestrant in post-menopausal advanced-breast cancer women previously treated with endocrine therapies has been largely studied. Fulvestrant proved efficacy at 250 mg both in the second and first-line settings [10, 11, 12, 13, 14, 15]. In a phase II neoadjuvant trial, fulvestrant, at the high- dose (HD) of 500 mg was associated with an increased biological activity compared to the dose of 250 mg, resulting in greater reductions of ER expression and inhibition ofof cell growth [16, 17, 18]. On this basis, a double-blinded, randomized, phase III study (CONFIRM) compared the two doses of fulvestrant and showed the superiority of the high dose [19, 20]. Confirmative evidence on the efficacy of fulvestrant 500 has come from other trials [21, 22, 23, 24]. Moreover, the FIRST trial has compared the activity of HD fulvestrant to Anastrozole as fist-line treatment for naïve hormone receptor-positive metastatic breast cancer patients. The study showed higher efficacy of fulvestrant [25, 26], which was further confirmed by a phase III randomized clinical trial of fulvestrant 500 mg compared to Anastrozole as first-line hormonal treatment in patients with hormone-receptor positive metastatic breast cancer [27]. Since most of the patients enrolled in randomized clinical trials are selected, we performed a multicenter observational prospective trial to evaluate the efficacy of fulvestrant in advanced breast cancer patients treated in routine practice.

## RESULTS

**Figure 1:**
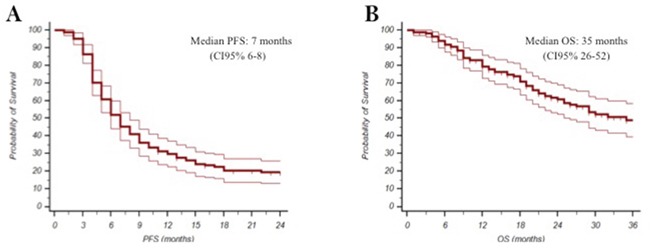
Kaplan-Meyer plot for Progression Free Survival **(A)** and Overall Survival **(B)**.

**Figure 2:**
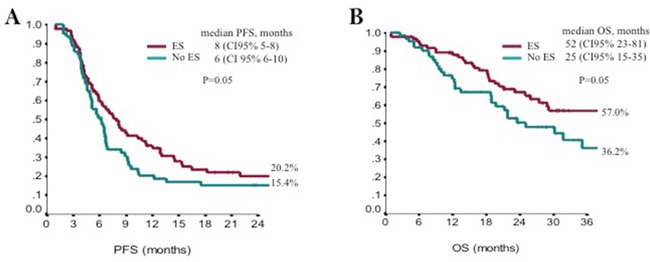
Kaplan-Meyer plot for Progression Free Survival **(A)** and Overall Survival **(B)** according to endocrine sensitivity (ES).

One hundred and sixty-three (N:163) consecutive eligible patients from 2010 to 2015 were enrolled at four Italian oncologic centers. Main patients’ characteristics were reported in Table 1: median age was 68 years (range 35-87), ECOG PS was 0/1 in 95% of patients, adjuvant ET was administered in 75% of patients, 55% of them had received first-line ET (11 tamoxifen, 78 AIs), 30% had bone-only disease, 44% visceral disease, 52% had more than one site of disease, the expression of estrogen receptor/progesterone receptors was >50% in 78/50%. The majority (98.8%) of these patients received treatment as planned without delays or drug omission; 67% of patients received further therapy after fulvestrant. Safety analysis did not show relevant toxicity, no grade 3 or 4 toxicities were reported and no serious adverse events were observed (Table 2). All the events were manageable and main toxicities were: pain in the injection sites, asthenia, and arthralgia. Overall, CBR was reached in 61% of patients (95%CI 51-66), with a median duration of 10.8 months (CI95% 8.2-13.4). At a median follow up of 20 months (mo), median PFS was 7 months (95%CI 6-8), and median OS was 35 months (95%CI 26-52) (Figure 1A and 1B). Endocrine sensitive (ES) patients had better PFS and OS compared to non-endocrine sensitive women (non-ES). Median PFS was 8 (95% CI 5-8) months in ES vs 6 months (CI 95% 6-10) in non-ES (p=0.05), and median OS was 52 months (95CI% 23-81) in ES vs 25 months (95% CI 15-35) in non-ES (p=0.05) (Figure 2A and 2B). In multivariate analysis, visceral involvement, endocrine sensitivity and previous ET were prognostic factors for PFS, whereas endocrine sensitivity and metastasis at diagnosis were prognostic factors for OS. Estrogen receptor expression >50%, no more than 1 site of metastasis and no previous ET for advanced disease were predictive of CBR (Table 3).

**Table 1:** Characteristics of patients

Number of patients	n=163	%
**Median age (yrs)**	68 (range 35-87)	
≤65	70	42.9
> 65	93	57.1
**ECOG Performance status**		
0	118	69.8
1	37	24.5
2	8	5.8
ER positive/PR positive	141	86.5
ER positive/PR negative	22	13.5
Her 2 negative	145	91.8
Her 2 positive	13	8.2
**Metastatic sites**		
Bone	50	30.7
Node	47	28.8
Visceral	72	44.2
Brain	3	1.8
Other	13	8.0
**Number of sites of disease**		
1	78	47.9
>1	85	52.1
**Previous endocrine therapy**		
* Adjuvant*	120	73,6
Tamoxifen	63	39.0
Aromatase inhibitors	75	46.0
* Advanced first line ET*	89	54.6
Tamoxifen	11	6.7
Aromatase inhibitors	78	41.7
**Setting of Fulvestrant administration**		
1^st^ line setting (naïve)	9	6
Fulvestrant after adjuvant ET	58	35
Fulvestrant after ET for advanced disease	81	50
1^st^ line maintenance after CT for advanced disease	15	9
**Endocrine sensitive (ES)**	90	55.2

**Table 2:** Adverse events

	Grade 1	
	n	%
Pain	72	44.2
Asthenia	29	17,8
Arthralgia	24	14.7
Headache	15	9.2
Nausea/vomiting	4	7.5
Hyperlipidemia	4	2.5
Flushes	3	1,8
Constipation	1	0.6
Diarrhea	1	0.6

**Table 3:** Multivariate regression analysis

PFS	HR	IC95%	P
Visceral(yes vs no)	1.52	1.06-2.18	0.022
Previous endocrine therapy(>1 vs 1)	1.88	1.28-2.76	0.001
Endocrine sensitivity(no vs yes)	1.66	1.14-2.43	0.009
**OS**			
Number sites of disease(>1 vs 1)	2.29	1.37-3.85	0.002
Endocrine sensitivity(no vs yes)	1.61	0.98-2.64	0.059
Metastasis at diagnosis(no vs yes)	1.98	1.11-3.55	0.021
**CBR**	**OR**		
Estrogen receptor(>50% vs <50%)	3.49	1.30-9.38	0.01
Site of metastasis(1 vs >1)	2.21	1.08-4.50	0.03
Previous ET for advanced disease(no vs yes)	2.24	1.1-4.58	0.03

## DISCUSSION

Fulvestrant represents an ET option for endocrine sensitive breast cancer in the post-menopausal setting. The strength of this drug is strictly related to the high compliance ensured by the intramuscular injection that allows its regular administration. This avoids the uncertainty of assumption which may affect the efficacy of any orally administered drug. The low incidence of adverse events further encourages fulvestrant use. The dose of fulvestrant used in our population (500 mg) was superior to the 250 mg used in the early trials comparing fulvestrant to AIs and tamoxifen, and represents the currently approved dose. Recently, a network meta-analysis has showed the efficacy of 500 mg fulvestrant in terms of OS over the 250 mg dose and megestrole acetate [28]. Although clinical trials provide high quality data about the efficacy and safety of drugs, additional details from the “outside trial” setting could help physicians in the daily practice. Consistent evidence in support of fulvestrant safety and efficacy in the clinical practice have come from recent studies. In the retrospective trial from Ishida and co-authors, 117 patients were treated with HD fulvestrant. In this cohort, CBR was 42% and median time to progression (TTP) was 6 months; previous endocrine sensitivity and absence of liver metastasis correlated with TTP [29]. In our prospective trial, we evaluated the efficacy of fulvestrant in the clinical daily practice. Our results from the present study confirm fulvestrant efficacy, with a CBR which fairly compares with data from randomized clinical trials. As expected, ES patients showed better outcome compared to non-ES patients, while patients with visceral disease had the worst outcome. Earlier use of fulvestrant was associated with better response. In addition, patients with an estrogen receptor percent expression higher than 50% seems to respond better than patients with lower percent expression. Within the highly-expressing subset of patients, the ability of Fulvestrant HD to exert ER-down regulation is mainly due to the blockade of of ER-mediated transcription and acceleration of ER-degradation through ubiquitin proteolysis [30]. This prospective trial has some limitations, it is a prospective evaluation of patients who were not selected upon strictly applied inclusion/exclusion criteria and treated as per clinical practice. This leads the way to a number of potential biases. Nonetheless, results from our analysis do not differ from those reported in the literature, thus confirming the effectiveness of this treatment even in unselected population outside of clinical trials. New treatments for the endocrine sensitive breast cancer setting are now under development. Ongoing trials are currently exploring the associations of fulvestrant or AIs with cyclin-dependent kinases 4 and 6. Results from the PALOMA-2, that compared treatment with palbociclib and letrozole with letrozole alone in the first-line setting for endocrine naïve patients, showed impressive improvement of median PFS for the palbociclib and letrozole association (24.8 months compared to 14.5 months of the letrozole group). However, data on OS are not available yet [31]. The PALOMA-3 trial compared palbociclib and fulvestrant with fulvestrant alone in pretreated patients. Median PFS for the experimental arm was 9.2 months compared to 3.8 months for the control group. Other cyclin-inhibitors are under investigation and the upcoming results will clarify the role of their association with the currently available endocrine therapies [32, 33, 34]. The addition of the CDK4/6 inhibitor ribociclib to letrozole showed a 44% improvement of the PFS of postmenopausal advanced breast cancer women treated in the first line setting [35]. Moreover it will be of interest the role of fulvestrant in estrogen receptor (ESR1) mutant breast cancers. Retrospective analyses of ESR1 status were performed in the SoFEA and PALOMA 3 trials. Patients with ESR1 mutated tumors seemed to respond better to fulvestrant or fulvestrant plus palbociclib [36, 37]. The results obtained from the present analysis show that fulvestrant remain an optimal choice in first-line as well as in second line following prior hormone therapy. Fulvestrant 500 mg has consistently showed to be a safe and active treatment and has confirmed its efficacy also in the daily clinical practice. Treatment was very well tolerated. A high estrogen receptor expression (above 50%) seems to correlate to a higher CBR. Endocrine sensitivity surely has a major impact on treatment outcome and, as expected, patients who had received first-line ET for advanced disease prior to fulvestrant had worse outcome, including lower CBR. To our knowledge, this study represents the first prospective trial, carried out in the clinical practice setting to confirm the efficacy and safety of fulvestrant HD therapy. In this view, our results may add useful information to the management of endocrine sensitive breast cancer patients. From a cost-effective point of view, fulvestrant HD was compared to the 250 mg dose and to the generic nonsteroidal aromatase inhibitors (anastrozole and letrozole); an increase of costs were observed and this can be a critical issue if we consider the financial sustainability of the national health system in some disadvantaged regions. However, certain subgroups of patients may benefit from fulvestrant as a treatment option [38, 39]. The recent results from the FALCON trial confirmed [27] the efficacy of fulvestrant in the first line treatment and added a new opportunity to the first-line options available for treatment of naïve endocrine sensitive breast cancer patients.

## MATERIALS AND METHODS

Eligible patients were women with advanced endocrine sensitive breast cancer, suitable to receive endocrine therapy with fulvestrant. Patients were included if having experienced relapse while on or after the completion of adjuvant endocrine therapy, or presenting with *de novo* advanced disease, or if previously treated with either an antiestrogen or an aromatase inhibitor in first-line. Patients who received fulvestrant as maintenance therapy after chemotherapy for advanced disease were also included. Endocrine sensitive patients were defined as follows: patients who progressed after a least 24 months from the completion of adjuvant endocrine therapy (ET) or after at least than 24 weeks of endocrine treatment for advanced disease. The lack of endocrine sensitivity was defined as disease progression while on or within two years from the completion of adjuvant ET or patients who progressed within the first 24 weeks of endocrine treatment for advanced disease. Main exclusion criteria were: extensive visceral involvement, visceral crisis, central nervous system metastases not adequately controlled, Eastern Cooperative Oncology Group Performance Status (ECOG PS) >2. Fulvestrant 500 mg was given as two 5-mL intramuscular (IM) injections, on days 0, 14, and 28 for the first months and every 28 days thereafter until progressive disease, unacceptable toxicity or withdrawal of informed consent. Response was evaluated as per RECIST 1.1 criteria every 12 weeks from baseline until progressive disease. Adverse events were monitored monthly and classified with the National Cancer Institute Common Toxicity Criteria v.4.0 (NCI-CTC 4.0). The primary endpoint was clinical benefit rate (CBR) defined as the sum of complete responses (CR), partial responses (PR) and stable disease (SD) for at least 24 weeks. Secondary endpoints were progression free survival (PFS) and overall survival (OS).

### Statistical analysis

Descriptive statistics were used to summarize pertinent study information. The Chi-Square or Fisher Exact tests were used to estimate associations between categorical variables. Odds Ratio (OR) and the 95% confidence intervals (95% CI) were estimated for each variable of interest in univariate regression models. The variables tested by univariate analysis were age (<65 vs ≥65 years), performance status (0-1 vs 2), expression of estrogen and progesteron receptor (≥50% vs <50%), Her-2 positivity (score 3+ vs others), Ki-67 value (<20% vs >20%, sites of disease (visceral vs other), metastasis at diagnosis (yes vs no), previous endocrine therapy (aromatase inhibitors vs antiestrogen), number of previous line of treatment (first-line vs ≥2), endocrine sensitivity (yes vs no). A multivariate logistic regression model was developed using stepwise regression (forward selection, enter limit and remove limit, p=0.10 and p=0.15, respectively) to identify independent predictors of CBR. Survival estimates were computed and compared by the Kaplan-Meier product-limit and log-rank test. The Hazard Ratio (HR) and 95% confidence intervals (95% CI) were estimated by using Cox univariate models. The included variables for PFS and OS were age (<65 vs ≥65 years), performance status (0-1 vs 2), expression of estrogen and progesteron receptor (≥50% vs <50%), Her-2 positivity (score 3+ vs others), ki 67 percent expression (<20% vs >20%, sites of disease (visceral vs other), metastasis at diagnosis (yes vs no), previous endocrine therapy (aromatase inhibitors vs antiestrogen), number of previous line of treatment (first-line vs ≥2), endocrine sensitivity (yes vs no). A multivariate Cox proportional hazard model including clinical-pathological features and details on treatment was developed using stepwise regression (forward selection, enter limit and remove limit, p=0.10 and p=0.15, respectively), to identify independent predictors of PFS and OS. The SPSS software (SPSS version 21.0, SPSS Inc., Chicago, Illinois, USA) was used for all statistical evaluations. The sample size was calculated based on an estimated CBR of 45% observed in our previous fulvestrant trial [19]. Effectiveness measures included PFS duration, defined as the time elapsed between the date at fulvestrant start and disease progression [PD] or death, and OS duration, defined as the time window between the begin of fulvestrant and death or censoring. Disease staging was performed as per clinical practice with physical examination and diagnostic techniques for radiologic imaging. Fulvestrant treatment was continued until disease progression, unacceptable toxicities, and patient consent withdrawal. Subsequent lines of therapy were at the discretion of the investigators. The ethics committee of each participating institution approved the protocol. Patients were treated according to the Helsinki declaration and a written informed consent was secured from each patient before study entrance.
